# Mixed-methods approach to evaluate an mHealth intervention to increase adherence to triage of human papillomavirus-positive women who have performed self-collection (the ATICA study): study protocol for a hybrid type I cluster randomized effectiveness-implementation trial

**DOI:** 10.1186/s13063-019-3229-3

**Published:** 2019-02-26

**Authors:** Silvina Arrossi, Melisa Paolino, Liliana Orellana, Laura Thouyaret, Racquel E. Kohler, Kasisomayajula Viswanath

**Affiliations:** 1 Centro de Estudios de Estado y Sociedad/Consejo Nacional de Investigaciones Científicas y Técnicas Sociedad, Sánchez de Bustamante 27, 1193 Buenos Aires, Argentina; 2 Centro de Estudios de Estado y Sociedad/Consejo Nacional de Investigaciones Científicas y Técnicas, Sánchez de Bustamante 27, 1193 Buenos Aires, Argentina; 3 Biostatistics Unit, Faculty of Health, Deakin University, Geelong, Australia; 4 Programa Nacional de Prevención de Cáncer Cervicouterino/Instituto Nacional del Cáncer (Argentina), Julio A. Roca 781, Piso 9, 1067 Buenos Aires, Argentina; 5 Department of Social and Behavioral Sciences, Harvard T.H. Chan School of Public Health, Harvard University, Boston, USA

**Keywords:** mHealth, Self-collection, HPV, Community health workers, Implementation

## Abstract

**Background:**

Cervical cancer is one of the leading causes of cancer death among women worldwide, with more than 85% of cases occurring in low- and middle-income countries. Human papillomavirus (HPV) screening allows for self-collection with the potential to increase coverage, but still requires triage to identify which HPV+ women need diagnostic and treatment procedures. However, achieving high levels of triage adherence can be challenging, especially among socially vulnerable women. This paper describes the ATICA protocol (Application of Communication and Information Technologies to Self-Collection, for its initials in Spanish), aimed at evaluating the implementation strategy and the effectiveness of a multi-component mobile health (mHealth) intervention to increase adherence to triage among women with HPV+ self-collected tests.

**Methods:**

We will use an effectiveness-implementation hybrid type I trial with a mixed-methods evaluation approach. A cluster randomized trial design including 200 community health workers (CHWs) will evaluate whether the mHealth intervention increases adherence to triage among HPV+ women who self-collected at home during a CHW visit within 120 days after a positive result. The intervention includes an initial mobile phone text message (SMS) alert and subsequent reminders sent to HPV+ women. For those who do not adhere to triage within 60 days of a positive HPV test, an email and SMS will be sent to the CHWs to promote contact with these women during home visits.

We will use the Consolidated Framework for Implementation Research (CFIR) as an organizing and analytic framework to evaluate the implementation of the intervention while also drawing on Reach, Effectiveness, Adoption, Implementation, and Maintenance (RE-AIM). We will conduct a self-administered, semi-structured survey of CHWs, semi-structured interviews with local health authorities, and a survey of HPV+ women. Combining both qualitative and quantitative data will provide rich insights into local implementation challenges and successes.

**Discussion:**

Findings from the implementation evaluation will be applicable to programs that use or are planning to incorporate HPV self-collection and/or mHealth interventions in different settings and countries. This innovative study will also serve as a model for using implementation science in the region.

**Trial registration:**

ClinicalTrials.gov, NCT03478397. Registered on 20 March 2018.

**Electronic supplementary material:**

The online version of this article (10.1186/s13063-019-3229-3) contains supplementary material, which is available to authorized users.

## Background

Cervical cancer (CC) is one of the leading causes of cancer death among women from low- and middle-income countries (LMIC), where 85% of worldwide CC cases occur [1]. Human papillomavirus (HPV) DNA testing is a highly effective screening method [2] and allows women to self-collect samples [3, 4], dramatically reducing barriers to screening. In a self-collection screening program, triage of abnormal screening results is a key step in identifying HPV+ women who will need further diagnostic and treatment procedures. While several triage methods are available for detecting precancerous lesions, cytology has been validated in several randomized trials and is part of the screening policy of several European and Latin American countries (LAC) [5]. Cytology involves HPV+ women attending health centers for triage to determine appropriate follow-up. However, adherence to triage and treatment is a widespread problem for CC programs in Latin America [6], especially among women who are not regular health system users [7]. Efficacious interventions aimed at improving adherence to triage are needed.

In Argentina, one of the first countries in the world to implement HPV testing as the primary screening method [8], adherence to triage of HPV+ women with self-collected tests is a challenge [9]. During the first year of programmatic scaling-up of self-collection in the province of Jujuy, triage was 30% in the 4 months after screening. After a significant effort by community health workers (CHWs) to contact women at their homes, adherence increased to 77% by 12 months [9]. Studies that have analyzed adherence to different follow-up steps after abnormal cytology [10–12] show that one of the key issues is the delivery of test results. In a study carried out in Argentina, not receiving results was one of the most reported barriers to follow-up by women who were not adherent to diagnosis and treatment after a positive Papanicolaou (Pap) result [12]. However, home visits by CHWs to all HPV+ women as a public health strategy is difficult to sustain as a high proportion of all screened women would need to be contacted (around 13%) [8]. A mobile health (mHealth) intervention has the potential to increase triage adherence among HPV+ women without being heavily dependent on scarce human resources.

Using mobile phone text messages (SMS messages) as reminders has proven to be effective in a variety of settings and for different health problems (e.g., noncommunicable diseases and AIDS [13–18]). SMS messages are useful for reminding patients about medication adherence (e.g., antiretroviral therapy and asthma treatment [13–15]), and to improve preventive and outpatient clinic attendance rates in many LMIC [16–19]. Similarly, mHealth tools targeted at providers, including CHWs, improve the quality of services they provide, most prominently through decision support and reminders [20–23]. SMS messages have advantages over other reminder systems, including that they can be sent to providers and patients and require less staff [13, 18]. SMS messages can enhance the link between patients and health services and increase adherence in primary care and gynecology care settings via reminders, counseling, or by addressing patient apprehensions [13, 16–18].

In this paper, we present the protocol of the ATICA study (Application of Information and Communication Technologies to Self-collection, for its initials in Spanish). This effectiveness-implementation hybrid type I trial aims to develop, test, and evaluate the implementation of an innovative multi-component mHealth intervention: SMS messages to women testing positive after HPV self-collection, and automated reminders to CHWs to visit and encourage HPV+ women who have not completed triage to attend health centers.

The proposed study is innovative by combining mHealth technologies with a personal contact with CHWs to increase adherence to triage of HPV+ women. There is increasing recognition that progress in complex health problems will come from integration of technological advances and social innovations [24]. A previous study carried out by our team to evaluate the effectiveness of self-collection offered by CHWs to increase screening uptake, the EMA (Evaluation of Self-collection Modality, for its initial in Spanish) study [25], showed that an integrated approach with synergy between two innovations, HPV self-collection and CHW work reorganization, can result in a real difference in CC control [25]. In the EMA study, women mentioned that the form and content of the messages transmitted by CHWs and the prior trust they had in them favored acceptance of self-collection [26]. Similarly, we expect that a multi-component intervention combining mHealth technologies with the work of CHWs will improve adherence to follow-up care, increase the effectiveness of the self-collection intervention, and accelerate reduction of disease burden.

## Methods

### Project overview

The specific aims of the study are:

1. To evaluate the effectiveness of a multi-component mHealth intervention to increase adherence to triage among women with HPV+ self-collected tests compared with usual care. We aim to increase adherence to triage of HPV+ women through SMS messages to HPV+ women, and automated reminders to CHWs to alert them to visit HPV+ women who have not responded to reminders and encourage follow-up.
2. To evaluate the implementation strategy and identify barriers and facilitators to implementation of the multi-component mHealth intervention.

The study design will follow the structure of an effectiveness-implementation hybrid type I trial [27] and will use a mixed-methods approach [28, 29]. We will use a hybrid type I design as proposed by Curran and colleagues [27] to galvanize the translation of efficacious treatments to enhance their public health impact. This hybrid design is appropriate for the proposed intervention because the following conditions are met: it has strong face validity that will support applicability to a new setting or population, there is at least indirect evidence for the intervention that will support applicability to a new setting or population, and there is minimal risk associated with the intervention [27].

We combine a cluster randomized trial to evaluate the effectiveness of a multi-component mHealth intervention with a mixed-methods approach involving quantitative and qualitative evaluations of the implementation using the Reach, Effectiveness, Adoption, Implementation and Maintenance (RE-AIM) [30] and Consolidated Framework for Implementation Research (CFIR) [31] frameworks. The mixed-methods design will allow a deep understanding of the reasons for success or failure of the mHealth intervention and also quantify the intervention impact on the outcomes [32]. ATICA will integrate quantitative and qualitative methods in multiple ways: a) formative research with women to develop the framing and content of SMS messages; b) a pragmatic cluster randomized trial to measure the effectiveness of the intervention in improving triage adherence among HPV+ women; and c) the acceptability of the strategy will be measured during the post-intervention phase through 1) a quantitative survey among CHWs, 2) semi-structured interviews with health authorities and health professionals, and 3) interviews with HPV+ women using a structured questionnaire to gather reasons for triage adherence/nonadherence.

We will use the RE-AIM [30] and CFIR [31] frameworks at all stages of the research process. The RE-AIM framework is particularly appropriate to assess the public health impact of interventions [30], and the CFIR is useful to systematically assess contextual factors that influence implementation and adoption [31].

### Hypothesis

Following the Health Belief Model [33, 34], we anticipate that the SMS messages will serve as specific cues to action and stimulate triage behavior. The CHW visit will be an additional cue to action to nonresponders, as well as an opportunity to provide counseling and support which will change women’s perceived benefits of and barriers to screening, as well as the perceived threat of CC. We expect both of these components to increase adherence to triage in the intervention group.

### Setting

The research will take place in the province of Jujuy, located in northwest Argentina (Jujuy total population: 673,307; women: 51%; urban population: 85% [35]). The rate of mobile phone penetration was 82% of urban households in 2011, though this percentage has probably increased [36]. The CC mortality rate in Jujuy is 10.6 per 100,000 women (2012–2014). The public health sector in Argentina includes a network of public hospitals and primary health care (PHC) centers which mostly provide care to people not covered by the social security sector (informal economy workers and unemployed people). For the uninsured, health services are provided free of cost. In Jujuy, the PHC system integrates approximately 700 paid full-time CHWs who twice yearly visit approximately 110,000 households for health-related tasks such as immunization and promoting maternal and child health. HPV testing was introduced in 2012 through the Jujuy Demonstration Project [8]; since then, it has become the primary screening test and is available to all women aged 30 and over at public health institutions. The provincial program on CC prevention uses the national screening information system (SITAM, for its initials in Spanish) [37]. SITAM registers all screening, diagnosis, and treatment events of women screened in the public health system and works as an online medical record. Results of HPV tests and Pap smears are immediately registered in SITAM to generate a diagnosis form, which is instantly available to providers at public health establishments with an online terminal. Since 2014, HPV self-collection is available and offered during the CHW routine home visits, and those women who accept the offer perform self-collection at home. Women who opt for self-collection are instructed to go to the health center within 30 days to pick up the results. In 2014 and 2015, approximately 38% of all women aged 30 years and over who were screened through the public health sector did it through self-collection [38].

### Intervention development

#### Formative research

A minimum of four focus groups discussions (FGD) will be carried out to explore women’s opinions about how to frame SMS messages and their content. All FGDs will have 6–12 women stratified by age (30–50 and 51 or more years old) and location (urban/rural). Participants will be recruited by CHWs during routine health rounds, a recruitment strategy which we have used and found to be effective [26]. CHWs and participants who participated in the formative research will not be eligible to take part in the cluster randomized trial. With prior authorization of the participants, each FGD will be audio-recorded to facilitate further analysis. We will use both audio and textual transcripts for the analysis. The Health Belief Model will guide our qualitative analysis to help identify and operationalize the responses and reactions of women to SMS messages, allowing us to also identify perceived benefits and barriers to triage as well as cues to action [33, 34]. Feedback from FGDs will be used to develop SMS messages which will be validated and pilot tested.

#### Development of the automated messaging system (AMS)

We will develop an AMS to send SMS messages and e-mails. The AMS will connect with SITAM to identify women to whom the SMS messages will be sent.

#### SMS message validation and pilot test

The validation process will include a sample with at least 10 women aged 30 years old or above. Women will be recruited in public PHC centers and will be asked to provided signed informed consent. They will receive the SMS proposed for the intervention to their phones and then trained interviewers will administer an ad-hoc individual questionnaire using face-to-face interaction. The questionnaire will include open- and closed-ended questions. The design of the questionnaire will follow the procedures and purposes of cognitive interviews, adapting the instruments used by other researchers [39, 40]. Different techniques will be combined to evaluate the validity of contents and procedures, and thus ensure that participants understand the instructions and respond in the same way using similar criteria [41–43].

After the validation process, we will carry out a pilot test. Five CHWs (not included in the sample) will be recruited and trained. During their routine home visits, they will invite eligible women to participate in the study. Upon registration of HPV test results into the SITAM database, the AMS will send the corresponding messages. We will then carry out telephone interviews using the ad-hoc questionnaire to test the reception of SMS messages, and the perception of women regarding its content and objective.

### Cluster-randomized trial

#### Participants

##### CHW eligibility

All CHWs (clusters) of the Jujuy province who offer self-collection and have a minimum of 26 potentially eligible women to participate in the study will be eligible (approximately 500).

##### Women eligibility

All women living in a house visited by a selected CHW will be invited to participate if they satisfy the following inclusion criteria:

1. aged 30 years or older;
2. have performed HPV self-collection offered by CHWs during the current routine home visit;
3. are competent enough to understand the consent form;
4. are able to communicate with CHWs;
5. are able to provide a mobile phone number.

#### Intervention

**Fig. 1:**
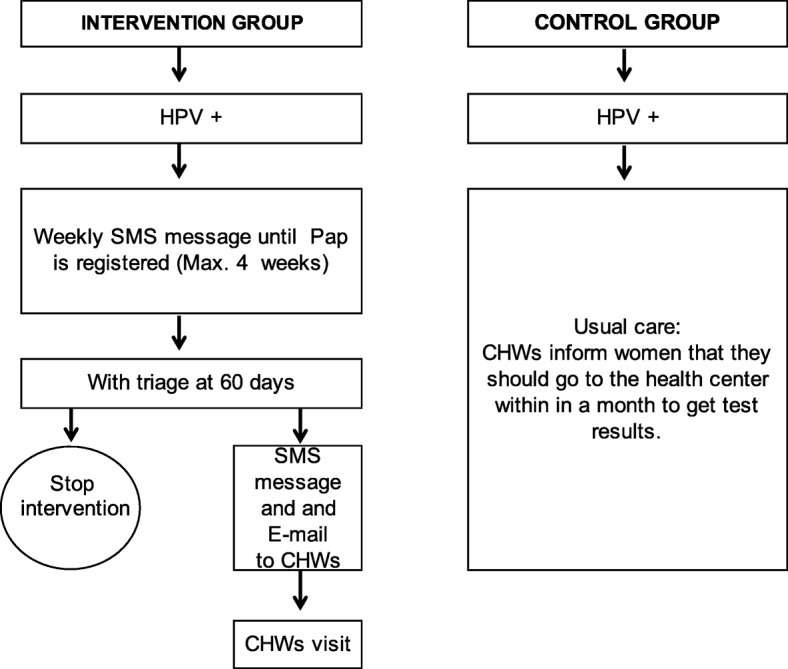
Flow diagram of the mHealth intervention. CHW community health worker, HPV human papillomavirus

Women with positive self-collected tests will receive a multi-component intervention (Fig. 1). Upon registration in SITAM of the HPV result at the laboratory, they will receive a weekly SMS message for 4 weeks, notifying them that the test results are available and that they should go to the health center. Messages will be stopped if a Pap result is registered in SITAM.

In addition, CHWs will receive an e-mail and SMS message sent through the AMS to visit those HPV+ women who, at 60 days since the HPV result, have not attended triage. CHWs will visit nonadherent HPV+ women within 15 days of being notified for an in-person reminder and will provide counseling about the importance of triage. The subgroup of HPV+ women visited by CHWs are given 30 days to attend health centers for triage. This timeline is in agreement with the screening protocol of the provincial screening program.

Negative HPV women will receive one SMS message stating that results are available at the health center.

Women with HPV+ self-collected tests participating in the control group will receive usual care as described in the Settings section.

All HPV tests and Pap smears will be processed at the provincial laboratory (average time 15 days between smear collection and report) and the results input into SITAM.

**Fig. 2:**
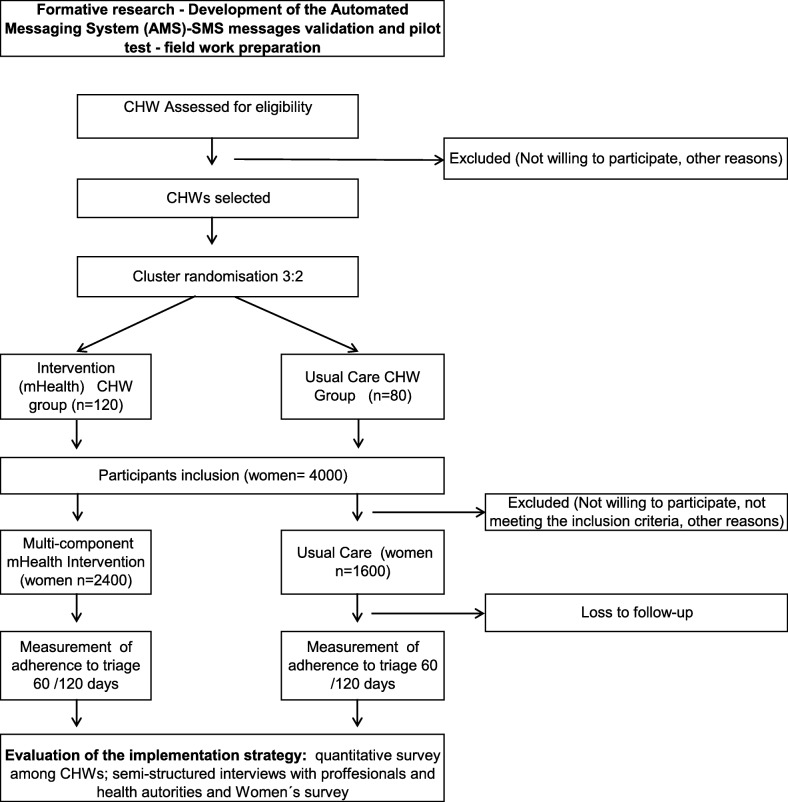
Flowchart of the ATICA cluster randomized trial. CHW community health worker

**Fig. 3:**
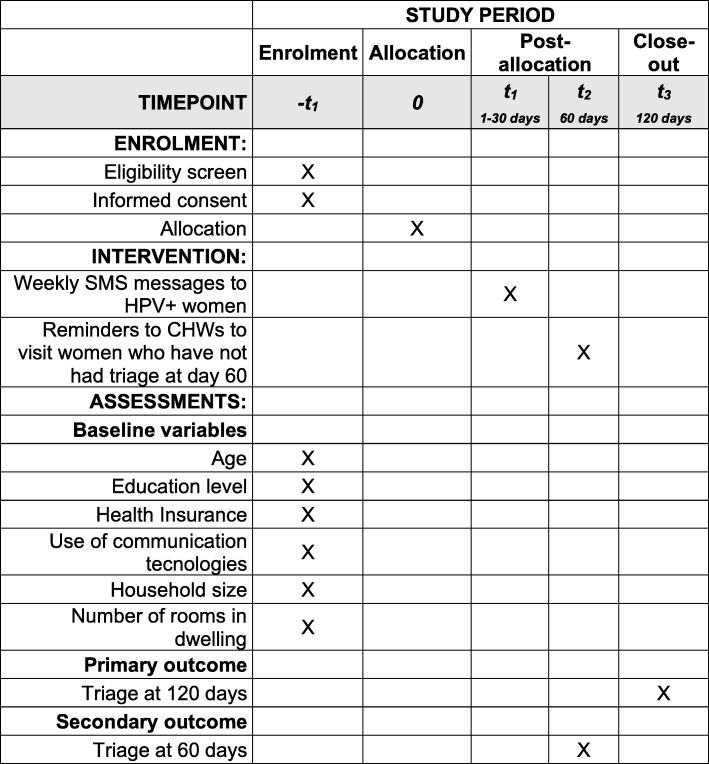
Schedule of enrolment, interventions, and assessments for the ATICA study. CHW community health worker, HPV human papillomavirus

Figure 2 offers a CONSORT flow diagram depicting trial procedures, Fig. 3 shows the schedule of enrolment, interventions, and assessments [44], and Table 1 shows the timeline of the ATICA project. The protocol presentation also follows the SPIRIT 2013 checklist [44] (Additional file 1).

**Table 1:**
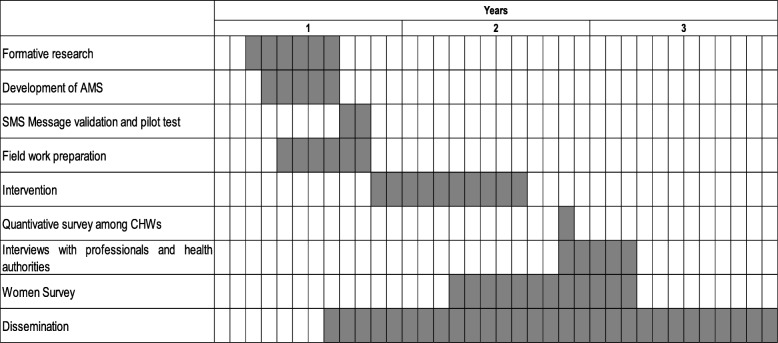
Timeline of ATICA Study

*AMS* automated messaging system, *CHW* community health worker

#### Outcome measures

The primary outcome of the trial is the percentage of HPV+ women with triage 120 days after the HPV result has been uploaded to SITAM, which measures the overall effect of the multi-component intervention (i.e., SMS message sent to women and SMS message and e-mail sent to CHWs to visit women with no triage at day 60). The secondary outcome is the percentage of HPV+ women with triage 60 days after HPV test results have been uploaded to SITAM, which measures the individual effect of the SMS messages sent to women (before CHWs receive prompts to visit nonadherent HPV+ women).

#### Sample size and power considerations

We expect to collect information on approximately 400 HPV+ women (240 in the mHealth (MH) group and 160 in the usual care (UC) group). For the primary outcome (triage percentage at 120 days), the target sample size will have 97% power to detect a 20% absolute difference between the two groups when the UC group has a 30% triage (two-sided test, alpha = 0.05, intraclass correlation coefficient (ICC) 0.10). For the secondary outcome (triage percentage at 60 days) under the same assumptions, the proposed sample size will have 90% power to detect a 10% absolute difference between groups when the UC group has a 15% triage by day 60.

Power calculations were performed with PASS version 14 under very conservative assumptions: 1) the average number of HPV+ women per CHW was two (in fact, from the EMA trial [25] for CHWs with 13 or more women with self-collected tests, the average number of positive women was 4.1); and 2) an ICC of 0.10.

#### CHW and women recruitment

CHWs will be randomly selected and invited to participate in the study by the research team. All women living in households visited by a participating CHW satisfying the eligibility criteria will be invited to participate in the study during their routine home visits.

For the MH group, once women have performed HPV self-collection, CHWs will screen for eligibility criteria, including the use of a mobile phone. If the woman does not have a personal phone, she will be asked if she is willing to be contacted and receive SMS messages on a shared phone. Consent to participate will also mean that women agree to be contacted via SMS messages, even if phones are shared by several family members, and potentially in person again by the CHW. CHWs will explain the sequence and content of SMS messages, how participants should proceed after receiving the SMS messages, and that they might come back for a personal visit if they do not complete triage.

For the UC group, once women agree to participate in the study, CHWs will provide UC counseling using routine programmatic materials [45, 46] and information using standard provincial protocol and materials. They will inform women that they should go to the health center within a month to get their HPV test results.

#### Randomization

After classifying them into four groups according to gender and urban/rural setting, a stratified sample of 240 CHWs will be randomly selected with allocation proportional to strata. We expect a minimum of 200 will be enrolled. Enrolled CHWs will be randomly allocated to the MH or the UC group (3:2 ratio) using a computer-generated random number list generated by the study statistician. Allocation concealment is guaranteed because all CHWs will be assigned to the trial arms at the same time. Blinding of intervention and outcome assessments is not feasible due to the characteristics of the study.

The number of CHWs to be recruited was defined based on pragmatic considerations (120 CHWs in the MH group and 80 CHWs in the UC group). From our experience [25], this is a feasible number of CHWs that can be enrolled and trained in programmatic/routine conditions. We allocated a larger number of CHWs to the MH group to benefit the assessment of the implementation outcomes.

Based on PHC records and previous experience [25], we estimate that each CHW will enroll an average of 20 eligible women in a 10-month period. Thus, the unit of randomization is the CHW and the unit of observation is the woman.

We assumed an attrition rate of 20% for women due to problems with mobile phones (i.e., nonidentified number, unpaid phone bill, lack of signal, etc.), SMS messages not delivered, and/or other issues. Under a 13% prevalence of HPV+ in this population [8], we estimate that each CHWs will contribute an average of two HPV+ women.

#### Data collection

CHWs from both groups will use a specific form (Trial form) to collect data about women’s sociodemographic characteristics, inclusion/exclusion criteria, and tasks performed by the CHWs. Eligible women who do not agree to participate will be asked basic sociodemographic data (age, health insurance, education level, and reason for refusal) to allow comparison of enrolled and non-enrolled women.

Data on HPV testing, triage, diagnosis, and treatment will be imported from SITAM, where records will be linked through the ID number of women.

#### Data analysis

All analysis will be conducted on an intention-to-treat (ITT) basis; the effect of the multi-component mHealth intervention on the primary outcome will be assessed using a generalized estimating equations approach. The model (logit link and binomial distribution) will include the trial group as a fixed effect and CHWs as the clustering variable. Potential effect modifications due to the rural/urban location of the woman and CHW gender will be evaluated. Baseline sociodemographic characteristics of the women will be compared between groups using the same model with link and distribution selected depending on the variable. Factors showing clear unbalance between arms will be included in the model proposed above to adjust for potential confounding. The same approach will be used to estimate the intervention effect on the secondary outcome.

Analyses will be conducted using STATA 14.0 or SAS v.9.4.

#### Safety and monitoring

All SMS messages and e-mails will comply with security procedures according to NIH Human Protection Subject Guidelines [47]. The Principal Investigator (PI) will be responsible for monitoring adverse events and data quality and safety since this is a low-risk investigation. The plan for monitoring data quality and accuracy will include the following: data entry from the cluster randomized trial (CRT) will be performed through specific software that will include range and inter-item consistency checks. Entries outside of the expected range will not be permitted. Re-entry of 20% of questionnaires will be performed for quality control. The data cleaning process will actively search for errors in a planned way.

### Evaluation of the implementation strategy

We will use data from the CRT database and will also conduct a self-administered semi-structured survey of CHWs, semi-structured interviews with key stakeholders, and a survey of HPV+ women. RE-AIM and CFIR frameworks will guide the implementation evaluation (Table 2). The CFIR comprises 39 common constructs from published implementation frameworks and models and organizes these into five major domains [31]. In this study we will analyze the following domains: intervention characteristic (relative advantage, adaptability, complexity, cost); outer setting (patient needs and resources, external policies, incentives); inner setting (structural characteristics, tension for change, relative priority and available resources, access to knowledge and information); and characteristics of individuals (knowledge and beliefs about the intervention, perceived self-efficacy) [31].

**Table 2:** Measurements and data sources proposed for the implementation evaluation based on RE-AIM and CFIR

	Quantitative outcomes	Qualitative evaluation	Data source
Reach: representativeness of women reached by the intervention (quantitative data)	% of eligible women who accepted to participate in the study Sociodemographic information of participant/nonparticipant women		CRT database (Trial form)
Effectiveness in increasing women’s adherence to triage (quantitative and qualitative data)	Primary outcome: percentage of women with triage smears 120 days after test results are registered in SITAM	Reasons for adherence/nonadherence to triage	CRT database (SITAM) HPV+ Women Survey
Adoption by CHWs of the strategy of visiting HPV+ women after receiving SMS messages and e-mails^a^ Acceptability of the intervention by adopters	% of CHWs that visited at least one HPV+ woman after receiving the SMS message and e-mail % of CHWs that agreed with programmatic incorporation of the mHealth intervention	CFIR construct: knowledge and beliefs about the intervention; perceived self-efficacy	CRT database (Trial form + Automated Messaging System Monitoring Registry) Self-administered semi-structured survey of CHWs
Implementation of intervention activities according to protocol (quantitative data) Acceptability of the intervention by women (quantitative and qualitative data) Barriers and facilitators to implementing and administering the intervention (qualitative data)	% of randomized CHWs that participated in training % of SMS messages that reached a valid phone number % of e-mails that reached a valid e-mail address % of CHWs who sent confirmatory e-mails % of women who accept and are satisfied with the intervention	Woman level: experience and perception of the intervention; acceptability of SMS messages, pertinence of frequency, reception time, and content of SMS messages Stakeholder/CHW level: CFIR constructs: relative advantage; adaptability; complexity and cost. patient needs and resources and external policies and incentives Structural characteristics; tension for change, relative priority and available resources, access to knowledge and information	Automated Messaging System Monitoring Registry and Training Attendance List HPV+ Women Survey Semi-structured interviews with stakeholders and self-administered semi-structured survey of CHWs
Maintenance		Intention to incorporate the strategy	Interview with stakeholders

*CFIR* Consolidated Framework for Implementation Research, *CHW* community health worker, *CRT* cluster randomized trial, *HPV* human papillomavirus, *RE-AIM* Reach, Effectiveness, Adoption, Implementation, and Maintenance, *SITAM* national screening information system

Eight weeks after the CRT (intervention and measurement of primary outcome) is finished we will hold a workshop as a closing activity to present preliminary results to CHWs and health authorities. During this workshop, CHWs will be asked to complete an anonymous, self-administered semi-structured survey to evaluate their perspectives and acceptability of the intervention (see outcomes and qualitative evaluation in Table 2), and about barriers and facilitators of the intervention. We will administer the survey during the closing activity to assure a high response rate. Project staff members will emphasize that participation in the survey is anonymous and voluntary; all participating CHWs will sign consent forms. Those CHWs who are not able to attend this closing activity will receive the survey by mail and will be requested to send it to project staff in a blind envelope.

In the third year of the project we will conduct semi-structured interviews with stakeholders to evaluate their acceptability of the intervention and perspectives on how to build and enhance project sustainability (see qualitative evaluation in Table 2). Invited stakeholders will include directors and intermediate coordinators of the Minister and Secretaries of Health (the PHC Direction, the provincial Program on Cervical Cancer Prevention, etc.), and heads of gynecology services, and of the HPV laboratory. Constructs to be considered in the script will include: relative advantage, adaptability, complexity and cost; patient needs and resources, and external policies and incentives; structural characteristics; tension for change; relative priority; available resources; access to knowledge and information. Interviews with health authorities will also include questions to evaluate the potential for programmatic incorporation of the multi-component mHealth intervention to measure the maintenance dimension of RE-AIM. The sample size will be determined based on relevance and theoretical saturation [48]. We will select participants to guarantee that a wide range of responses is captured and to allow comparisons between them. To ensure we include enough stakeholders from relevant groups, we plan to conduct at least 20 interviews. With prior authorization of the participants, each interview will be audio-recorded.

Finally, we will carry out a structured questionnaire among all HPV+ women from the MH group (Women Survey) to evaluate their acceptability of the strategy, their perspectives about the implementation, and reasons for adherence and nonadherence to triage. All HPV+ women from the CRT intervention group (approximately 240 women) will be surveyed 5 months after their HPV test result (i.e., 1 month after measurement of the primary outcome), irrespective of their triage status. The questionnaire will include open and closed questions with dimensions related to women’s experiences and perceptions, including acceptability and relevance of SMS message frequency, receipt time, and content, the perceived effect of the multiple components of the intervention on their adherence, and reasons for adhering/not adhering to triage. The nominated list of these women and their contact details will be extracted from the CRT database. Trained interviewers will contact these women via telephone to make a home appointment to administer the questionnaire. The interviewers will be trained by the project staff; training will include formal aspects regarding applying the questionnaire as well as skills for developing a connection with the interviewees.

#### Analysis of women and CHW surveys

##### Quantitative data analysis

A descriptive analysis of each study dimension will be carried out using frequencies and percentages for each of the different survey variables. Outcomes will be compared across CHW sociodemographic characteristics using logistic regression and across women using generalized mixed linear models. Results will be presented as estimates and 95% confidence intervals. Analysis will be conducted using STATA v. 14.0 and/or SAS v. 9.4.

##### Qualitative data analysis

We will use CFIR as an organizing and analytic framework for Aim 2. We will use an iterative approach to analyzing qualitative data. Researchers will read, interpret, and identify themes and subthemes from data to develop a codebook [49]. To ensure coding reliability, two researchers will independently code all the interview transcripts. They will hold regular discussions to resolve disagreements and reach consensus, and will discuss uncertainties with a third team member (a specialist in qualitative research). After coding, thematic and content analysis will be conducted. We will use Atlas.ti to organize, analyze, and summarize common themes across types of participants.

## Discussion

The ATICA study is a cutting-edge multi-component mHealth intervention that combines mHealth technologies and primary health care to increase the effectiveness of CC prevention programs. The majority of the mHealth developments have targeted health problems such as hypertension, diabetes, tuberculosis, and HIV-AIDS [13–19]. To the best of our knowledge no previous use of these technologies in HPV self-collection screening programs has been published. By combining an mHealth intervention with CHW outreach we aim to improve adherence to triage, increase the effectiveness of the self-collection intervention, and accelerate reduction of disease burden. Key considerations of our study design include embedding the intervention in the existing health system, and conducting a pragmatic trial under programmatic conditions using existing human resources not requiring a complex reorganization of the CC prevention program already in place.

Combining mHealth technology with CHW outreach for CC prevention is also highly innovative. Using an automated system for communicating the availability of results immediately is a novel approach to inform HPV+ women about results because it reduces delay in result delivery and reinforces trust in the health system. Reminders will minimize forgetfulness, especially when women are busy with work or are away from home [50–52]. In addition, the proposed mHealth intervention will reinforce the privileged link that CHWs have with their communities to promote health information and behaviors. Despite being automated, these interventions are perceived to provide social support and reflect the concern of the health care providers [51]. Thus, mHealth technologies can facilitate and strengthen the relationship between CHWs and community women, creating a health-promoting environment. Furthermore, by prompting CHWs with SMS and email messages to make in-person visits to nonadherent HPV+ women, this intervention will provide a tailored component of social support to address barriers. The implementation evaluation will further show how an intervention can be planned, fielded, and evaluated using implementation science frameworks and tools, which is novel in LAC and in the CC prevention literature.

This study has several potential limitations. First, there is risk for contamination due to assigning CHWs who work in neighboring areas to different study groups, which could activate CHWs and women in the UC group. However, our prior work using CHW cluster randomization [25] suggests that this risk is low, and the effect of contamination is minimal. If such an effect occurs, we expect them to be moderate since meaningful improvements in adherence to triage require a systematic effort to address problems related to follow-up adherence which are unlikely to occur in the absence of a specific intervention. Second, it is not possible to blind the CHWs (clusters) or women in this study. However, the assessment of primary and secondary outcomes will be objective, as data on triage is extracted from SITAM. Third, there is potential for attrition due to poor cell phone network coverage or reception or gaps in the service. However, power calculations accounted for this type of attrition.

The results of this study will inform local and regional health systems and screening programs on the implementation of automated messaging systems to increase triage. If the intervention is effective, it could also be scaled-up to deliver results for clinician-collected HPV tests. Thus, the study design and approach can serve as a model of work, constituting an important advance in the use of implementation science in the region and for the field of cancer prevention. Ultimately, improved results delivery and increased triage adherence will increase the impact of HPV self-collection and help reduce unnecessary CC deaths.

## Additional file

Additional file 1: Standard Protocol Items: Recommendations for Interventional Trials (SPIRIT) Checklist: ATICA Study protocol. (DOC 120 kb)

## Abbreviations

AMS: Automated messaging system
CC: Cervical cancer
CHW: Community health worker
CRT: Cluster randomized trial
HPV: Human papillomavirus
ITT: intention-to-treat
LAC: Latin American countries
LMIC: Low- and middle-income countries
MH: mHealth
Pap: Papanicolaou
PHC: Primary health care
SITAM: National screening information system
SPIRIT: Standard Protocol Items: Recommendations for Interventional Trials
UC: Usual care
